# Is PD-L1 a consistent biomarker for anti-PD-1 therapy? The model of balstilimab in a virally-driven tumor

**DOI:** 10.1038/s41388-020-01611-6

**Published:** 2021-01-26

**Authors:** Joseph E. Grossman, Divya Vasudevan, Cailin E. Joyce, Manuel Hildago

**Affiliations:** 1 Agenus, Inc., Lexington, MA USA; 2 Division of Hematology and Medical Oncology, Weill Cornell Medicine, New York, NY USA

**Keywords:** Immunotherapy, Predictive markers

Immune-checkpoint inhibitors (ICIs) have substantially improved the outcomes of large subsets of patients across numerous malignancies. However, the lack of durable responses in many patients, added to high costs and the risk of adverse events, indicates a need for better biomarkers to guide patient selection. PD-L1 expression as measured routinely by immunohistochemistry (IHC), providing clinicians with a combined positivity score (CPS), microsatellite instability (MSI), and tumor mutational burden (TMB) have emerged as the most widely used and relevant biomarkers of response to ICIs. These biomarkers have variably succeeded or failed in predicting responders for different cancer types in adequately powered trials. Further, their prognostic and/or predictive roles are limited to specific settings.

PD-L1 expression by IHC is particularly controversial as an ICI biomarker. Indeed, multiple trials have shown efficacy of ICIs in patients with PD-L1 negative tumors and these agents are approved for treatment of PD-L1 negative cancers. Thus, in refractory or metastatic cervical cancer, the overall response rates (ORR) with nivolumab in PD-L1 positive and negative (CPS < 1%) patients were 2/10 (20%) and 1/6 (16.7%), respectively. Notably, the PD-L1 negative responder had a durable partial response that exceeded 24 months [1]. Pembrolizumab showed a 6% ORR in patients with PD-L1 negative (TPS < 1%) melanoma leading to an accelerated all-comer approval [2]. Furthermore, in third-line small cell lung cancer (SCLC), a 6% ORR in PD-L1 negative (CPS < 1%) patients also supported pembrolizumab’s accelerated all-comer approval [3]. Similar results were observed in second-line squamous cell carcinoma of the head and neck (SCCHN) in which a 6% ORR in PD-L1 negative (CPS < 1%) patients again supported pembrolizumab’s accelerated all-comer approval [4]. The ORRs in PD-L1 positive patients were 51% in melanoma, 36% in 3L SCLC, and 21% in SCCHN. In all cases, accelerated all-comer approval was granted despite lower ORRs in PD-L1 negative patients.

The recently reported interim data with the PD-1 inhibitor balstilimab (AGEN2034) in cervical cancer adds to the list of ICIs with activity in PD-L1 negative patients. Cervical cancer is almost always due to HPV infection and expresses a variety of foreign antigens. While much progress has been made in prevention, it remains a significant public health burden with limited therapeutic options for metastatic disease. Balstilimab (AGEN2034) is a next-generation anti-PD-1 therapy which demonstrated clinical benefit (14.3% overall response rate (ORR), 45.2% disease control rate (DCR)) as monotherapy in second-line cervical cancer patients (*n* = 143). Importantly, balstilimab demonstrated clinical benefit in patients with tumors that are both PD-L1 positive (ORR = 19%) and PD-L1 negative (CPS < 1%; ORR 10%) [5].

Although the basis for PD-1 inhibitors’ activity in PD-L1 negative tumors is incompletely understood, this consistent observation across many studies in multiple tumor types suggests that there is likely a biological basis for this phenomenon. PD-L1 can be an unreliable biomarker due to its dynamic expression, including a demonstrated propensity for increased expression with exposure to immunotherapy. Simply stated, patients who are negative at one point in time cannot be assumed to be negative later on. This phenomenon was exemplified in two studies that examined PD-L1 expression before and after cancer treatment. First, the prevalence of PD-L1 positivity on cancer and immune cells (TC or IC ≥ 1%) increased following platinum-based neoadjuvant chemotherapy in lung cancer patients (*N* = 63). Prior to treatment, 17.4% (11) and 15.9% (10) of patients expressed PD-L1 on tumor and immune cells, respectively. After treatment, 40.0% (25) and 71.4% (45) of patients expressed PD-L1 on tumor and immune cells, respectively [6]. Second, 65% (28/43) of melanoma patients treated with one cycle of nivolumab therapy exhibited upregulated PD-L1 expression as assessed by RNA-sequencing [7]. In cervical cancer lesions that are irradiated, this may also occur.

Mechanisms of PD-L1 upregulation include, but are not limited to, altered inflammatory signaling [8–10], oncogenic signaling [11–14], hypoxic signaling [15] post-translational modification [16, 17], and genetic alterations [18–20]. Given complex regulation, it is unsurprising to us that PD-L1 expression is heterogeneous within tumors and among different metastatic sites [21–23]. Together, dynamic temporal and spatial expression of PD-L1 limits interpretability of baseline PD-L1 testing. More relevantly, the method of measuring PD-L1 and the platform used may result in different PD-L1 values. Whilst only IHC has been used in routine practice and trials thus far, RNA-seq has the added advantages of being amenable to standardization and avoidance of interpretation bias. The ability of HPV to dysregulate such biomarkers has also been described.

For these reasons, the majority of anti-PD1 approvals have not required testing for PD-L1 positivity. Among 43 monotherapy approvals, (as of November 2020) only nine have required a PD-L1 positivity test. Of the nine exceptions, seven were linked to clinical trials for which there was a lack of evidence to support benefit or there was a lack of benefit for PD-L1 negative patients (Table 1). The C-700 trial adds to this list demonstrating the potential for balstilimab monotherapy to elicit responses in patients who are PD-L1 negative.

**Table 1:** Anti-PD-1 monotherapy approvals with PD-L1 companion diagnostic.

Clinical trials for which only PD-L1+ patients were treated	Keytruda in 1L NSCLC	KEYNOTE-024
	Tecentriq in 1L NSCLC	IMpower110
Clinical trials for which relatively few PD-L1− patients were assessed	Keytruda in chemotherapy-resistant cervical cancer • 16% PD-L1- pts (*N* = 16)	KEYNOTE-158 (Cohort E)
Clinical trials demonstrating limited benefit in PD-L1− patients	Keytruda in 2L+ esophageal • 1° endpoint met only in PD-L1 selected pts	KEYNOTE-181
	Keytruda in 1L SCCHN • No benefit vs cetuximab in PD-L1 unselected pts	KEYNOTE-048
	Keytruda in 1L urothelial cancer • Less benefit vs platinum-based chemo in PD-L1- pts	KEYNOTE-052 KEYNOTE-361
	Tecentriq in 1L urothelial cancer pts who are ineligible for platinum-based chemo • Less benefit vs platinum-based chemo in PD-L1- pts	IMvigor210 IMvigor130

## Conclusions

The need to predict and monitor patients’ responses to ICIs will make next-generation sequencing and other emerging biomarkers indispensable for identifying responders versus non-responders, as current biomarkers such as PD-L1 are inadequate. Currently, there is precedence for clinical benefit and even regulatory approval of PD-1 inhibitors with ~10% ORR in PD-L1 negative patients in multiple settings and excluding these patients from treatment may deprive them of a meaningful response to treatment. Taken together, and based on a head to head comparison, these data provide evidence to support the hypothesis that the assessment of PD-L1 status by IHC is an inadequate determinant of treatment benefit with anti-PD-1 therapies, including balstilimab.

In the future, we expect that therapeutic options for cervical cancer will include ICIs, chemotherapy, radiotherapy, surgery, small molecules and cell therapy, alone or in combination. For these patients, similar to the settings of neoadjuvant breast cancer and SCCHN, we have the ability to sample a patient’s tumor over time to better understand biomarker adaptations and complexities. Clinically unexpected response patterns, including late responders and pseudo- or hyper-progression, may complicate management as the underlying biology stems from complex dynamics between a drug, tumor and the host. We and others will continue to search for predictive clinically reliable biomarkers, as opposed to those based on the subjective expression of one single molecule. There is no doubt that such biomarkers will be dynamic, changing with time and context. While many academic and industry efforts have led us to biomarkers now in routine use in the clinic (PDL-1, TMB and MSI-status) as well as emerging biomarkers (neoantigen patterns, CD4/CD8 TIL infiltration rate, and Treg composition), the optimal role and application of each of these is still being elucidated. In recognition of this complexity, clinical trial task forces now understand the limitations of the measurement of a single biomarker, and future research will enable translating multi-dimensional biomarker discoveries into the clinic.
